# Identification of an IncHI5-like plasmid co-harboring *bla*_NDM−1_ and *bla*_OXA−1_ in *mcr-8.1*-positive *Klebsiella pneumoniae* isolate

**DOI:** 10.3389/fmicb.2025.1601035

**Published:** 2025-05-27

**Authors:** Xu Liu, Tingting Zhang, Zhiyang Yu, Shangshang Qin, Muchen Zhang, Yan Li

**Affiliations:** ^1^Department of Clinical Laboratory, The First Affiliated Hospital of Zhengzhou University, Zhengzhou, Henan, China; ^2^XNA Platform, School of Pharmaceutical Sciences, Zhengzhou University, Zhengzhou, Henan, China; ^3^Key Laboratory of Advanced Drug Preparation Technologies, Ministry of Education, Zhengzhou University, Zhengzhou, Henan, China

**Keywords:** *Klebsiella pneumoniae*, carbapenem-resistant, *mcr-8.1*, IncHI5-like, Genomics

## Abstract

The emergence of polymyxin-resistant carbapenem-resistant *Klebsiella pneumoniae* (CRKP) severely limits clinical treatment options and poses a significant threat to anti-infective therapy. In this study, we investigated the genetic characteristics of an IncHI5-like plasmid co-harboring *bla*_NDM−1_ and *bla*_OXA−1_ in an *mcr-8.1*-positive clinical CRKP isolate using a combination of MIC testing, conjugation experiments, bacterial whole-genome sequencing, and bioinformatics analyses. The ST626 CRKP strain KP19-2581, isolated from a human clinical infection, exhibited a multidrug-resistant (MDR) phenotype. Whole-genome sequencing revealed that the colistin resistance gene *mcr-8.1* was located on an IncFIA/IncFII plasmid, flanked by the conserved structure IS*903B*-*orf-mcr-8.1*-*orf*-IS*903B*. In addition, strain KP19-2581 carried a novel IncHI5-like MDR plasmid, designated pKP19-2581-367k-HI5-NDM1, which co-harbored the *bla*_NDM−1_ and *bla*_OXA−1_ genes. This plasmid contained two MDR regions, harboring a diverse array of resistance genes across multiple antibiotic classes. The dissemination of *bla*_OXA−1_ in variable region is related to the structure of class 1 integron, while IS*26* mediates the integration of *bla*_NDM−1_ on IncHI5-like plasmid. Notably, this is the first report of an IncHI5-like plasmid carrying both *bla*_NDM−1_ and two copies of *bla*_OXA−1_, along with multiple resistance genes and insertion sequences. Given its potential to acquire additional resistance determinants, this plasmid may serve as a reservoir for further antimicrobial resistance evolution, underscoring the urgent need for surveillance of IncHI5 plasmids to mitigate their clinical and epidemiological impact.

## Introduction

Carbapenem-resistant *Klebsiella pneumoniae* (CRKP) is globally widespread and represents a major threat to public health (Wyres and Holt, 2022; Yungyuen et al., 2021). The production of KPC-2, a type of serine-β-lactamases (SBL), is mainly responsible for carbapenem resistance in CRKP (Nordmann and Poirel, 2019; Meng et al., 2019; Meijing Shen et al., 2023). SBLs can be inhibited by various agents, including avibactam, making Ceftazidime-Avibactam (CZA) an effective therapeutic regimen for infections caused by KPC-producing *K. pneumoniae* (Krapp et al., 2017; Liu et al., 2022). However, the rise of MBL (metallo-β-lactamases) and SBL co-producing *K. pneumoniae* strains, particularly those harboring both *bla*_NDM−1_ and *bla*_KPC−2_, has significantly compromised the effectiveness of CZA. In these NDM and KPC co-producing CRKP strains, the *bla*_NDM−1_ and *bla*_KPC−2_ genes are carried by separate plasmids (Gao et al., 2020). Of note, our previous report identified both *bla*_KPC−2_ and *bla*_IMP−4_ genes were carried by a single conjugative IncHI5-like plasmid (Dong et al., 2023). The IncHI5 plasmid has been documented as a common vector for carrying single carbapenemase genes, such as *bla*_NDM_ or *bla*_IMP_. The co-harboring of two distinct types of carbapenemase further underscores the ongoing evolution of this plasmid type.

Polymyxins are considered the last line of defense against infections caused by carbapenem-resistant Gram-negative bacteria (Poirel et al., 2017). In China, back in November 2011, the plasmid-mediated polymyxin resistance gene *mcr-1* was initially detected in animals (Liu et al., 2016). Subsequently, additional *mcr* genes, including *mcr-2* through *mcr-10*, have been reported globally (Wang et al., 2020). The worldwide spread of *mcr* genes poses a serious menace to the final defense line of antibiotic resistance. Of particular concern is the increasing isolation of multidrug-resistant (MDR) *Klebsiella* strains that co-produce carbapenemases and MCR, which present substantial clinical challenges due to the limited treatment options available for these infections (Kaye et al., 2016; Mendes et al., 2018).

In this study, we report, for the first time, an IncHI5-like plasmid co-harboring *bla*_NDM−1_ and *bla*_OXA−1_ in an *mcr-8.1*-positive *K. pneumoniae* isolate, and comprehensively analyze its genetic characteristics. Through comparative genomics, we explored the genetic background differences of *bla*_NDM−1_ and *bla*_OXA−1_ on the IncHI5-like plasmid pKP19-2581-367k-HI5-NDM1, examining its integration mode to provide insights into the evolution of this plasmid. The co-existence of MBL genes, SBL genes, and the *mcr* gene in MDR strains poses a significant threat to public health. This emerging trend warrants close surveillance.

## Materials and methods

### Bacterial isolation, antimicrobial susceptibility testing and PCR identification

The CRKP isolate, designated KP19-2581, was obtained from the cerebrospinal fluid (CSF) culture of a 45-year-old female patient hospitalized in the neurological intensive care unit (NICU) at a teaching hospital of Zhengzhou University in 2019. The isolate was identified using the Vitek 2 system (bioMérieux, France) and 16S rRNA gene sequencing. Minimal inhibitory concentrations (MICs) were determined using the broth microdilution technique. Resistance breakpoints were interpreted according to Clinical and Laboratory Standards Institute guidelines (CLSI) guidelines (Clinical and Laboratory Standards Institute [CLSI], 2021), the MIC of antibiotics commonly used in clinical practice was determined. *E. coli* ATCC25922 was used as the quality-control strain. PCR was used to detect the existence of the colistin resistance gene *mcr* and the carbapenemase coding genes, including *bla*_NDM_, *bla*_IMP_, *bla*_VIM_, *bla*_OXA−48_-like and *bla*_KPC_. The primers were listed in Supplementary Table 1. This study was approved by the Ethics Committee of Zhengzhou University.

### Bacterial whole-genome sequencing (WGS) and bioinformatics analysis

Whole-genome DNA of *K. pneumoniae* KP19-2581 was extracted using the FastPure Bacteria DNA Isolation Mini Kit (Vazyme™, China) according to the manufacturer's instructions, and quantified using a Qubit 4 Fluorometer and NanoDrop (Thermo Scientific™, USA). The genomic DNA was subjected to both short- and long-read sequencing on the Illumina Hiseq 2500 platform with the PE150 strategy and Oxford Nanopore Technologies MinION platform. Following quality control and filtering with FastQC, hybrid *de novo* assembly was performed by combining Illumina short-read and Nanopore long-read data using Unicycler v.0.4.8 software (Wick et al., 2017). The assembled genomes were annotated using RAST (Overbeek et al., 2014). Open reading frame (ORF) prediction and annotation were carried out using Glimmer 3.02 (http://cbcb.umd.edu/software/glimmer/). Antibiotic resistance genes (ARGs) and multilocus sequence typing were performed using Kleborate (Lam et al., 2021). Plasmid replicons were identified using PlasmidFinder from the CGE website, with default parameters. ISs were identified using ISfinder with default parameters (Siguier et al., 2006). Sequence alignments for the genetic environments of *bla*_NDM−1_ and *bla*_OXA−1_ were performed using Brig and Easyfig (Sullivan et al., 2011; Alikhan et al., 2011).

### Conjugation experiments

Conjugation experiments were performed according to previously described methods. Briefly, ST11 *K. pneumoniae* strain HS11286YZ6 (hygromycin resistant) was served as the recipients to examine the transferability of the carbapenem resistance gene bearing IncHI5-like plasmids in CRKP. Cultured in Luria Bertani (LB) broth at 37°C for 6–8 h, the donor and recipient bacteria were mixed at a ratio of 1:1, and then diluted with fresh LB broth incubated at 37°C for 24 h (Sherburne et al., 2000). Subsequently, the mixture was plated on the LB agar containing double antibiotics and incubated at 37°C for 24 h. All suspected transconjugants which were confirmed by PCR amplification for carbapenem resistance genes and antimicrobial susceptibility test.

### Nucleotide sequence accession numbers

The complete nucleotide sequences of the KP19-2581 chromosome (CP1208772), along with plasmids pKP19-2581-367k-HI5-NDM1 (CP120875), pKP19-2581-207k-HIB (CP120874), pKP19-2581-101k-mcr-FII-FIA (CP120873), pKP19-2581-65k-R (CP120877), and pKP19-2581-3k-Col4401 (CP120876), have been deposited in GenBank.

## Results

### Phenotypic and genomic characteristics of CRKP KP19-2581

In 2019, clinical CRKP isolate KP19-2581 was recovered from the cerebrospinal fluid (CSF) culture of a 45-year-old female patient with meningitis in a teaching hospital of Zhengzhou University. Antimicrobial susceptibility testing showed KP19-2581 was resistant to all the β-lactams including meropenem and aztreonam, non-susceptible to colistin, but susceptible to amikacin and tigecycline (Supplementary Table 2). PCR results identified carbapenemase encoding genes *bla*_NDM−1_ and colistin resistance gene *mcr-8.1* in KP19-2581. Subsequently, Nanopore sequencing data analysis revealed KP19-2581, which was assigned to ST626, has one chromosome of 5,394,956 bp (CP120872) and five plasmids namely pKP19-2581-367k-HI5-NDM1 (CP120875), pKP19-2581-_−_207k-HIB (CP120874), pKP19-2581-101k-mcr-FII-FIA (CP120873), pKP19-2581-65k-R (CP120877), and pKP19-2581-3k-Col4401 (CP120876). The complete resistome is summarized in Table 1. Notably, co-occurrence of 18 resistance genes including *bla*_NDM−1_ and *bla*_OXA−1_ on a single mega-plasmid pKP19-2581-367k-HI5-NDM1, which prompted us to investigate the genetic features of this MDR plasmid. Conjugation assays demonstrated that the plasmid pKP19-2581-367k-HI5-NDM1 was non-conjugative and could not be transferred from the donor to recipient strains.

**Table 1:** Molecular characteristics of MDR *Klebsiella pneumoniae* strains KP19-2581.

**Characteristics**	**Size (bp)**	**G + C (%)**	**ORFs**	**Resistance genes**	**Plasmid type**
Chromosome	5,394,956	57	-	*bla*_SHV−187_, *fosA6, oqxB20, oqxA6*	-
pKP19-2581-367k-HI5-NDM1	367,802	48	417	*mph*(A), *aac(3)-IId*, *bla*_TME−1_, *acc(6′)Ib-cr*, *bla*_OXA−1_, *catB3, qacEdeltal1, sul1, sul2*, *ble*_MBL_, *bla*_NDM−1_, *arr-3, msr*(E), *mph*(E), *qnrS1*, *bla*_CTX−M−15_, *strB, strA*	IncHI5-like
pKP19-2581-207k-HIB	207,352	50	666	*bla*_CTX−M−15_	IncHIB
pKP19-2581-101k-mcr-FII-FIA	101,385	51	311	*mcr-8.1*	IncFII/FIA
pKP19-2581-65k-R	65,695	53	212	*aac(6′)Ib-cr*, *bla*_OXA−1_, *arr-3, catB3, qnrB4, sul1*, *bla*_DHA−1_, *mph*(A), *aph(3′)-Ia*	IncR
pKP19-2581-3k-Col4401	3,958	44	13	-	Col4401

### Characterization of a novel IncHI5-like plasmid co-harboring *bla*_NDM−1_ and *bla*_OXA−1_

**Figure 1:**
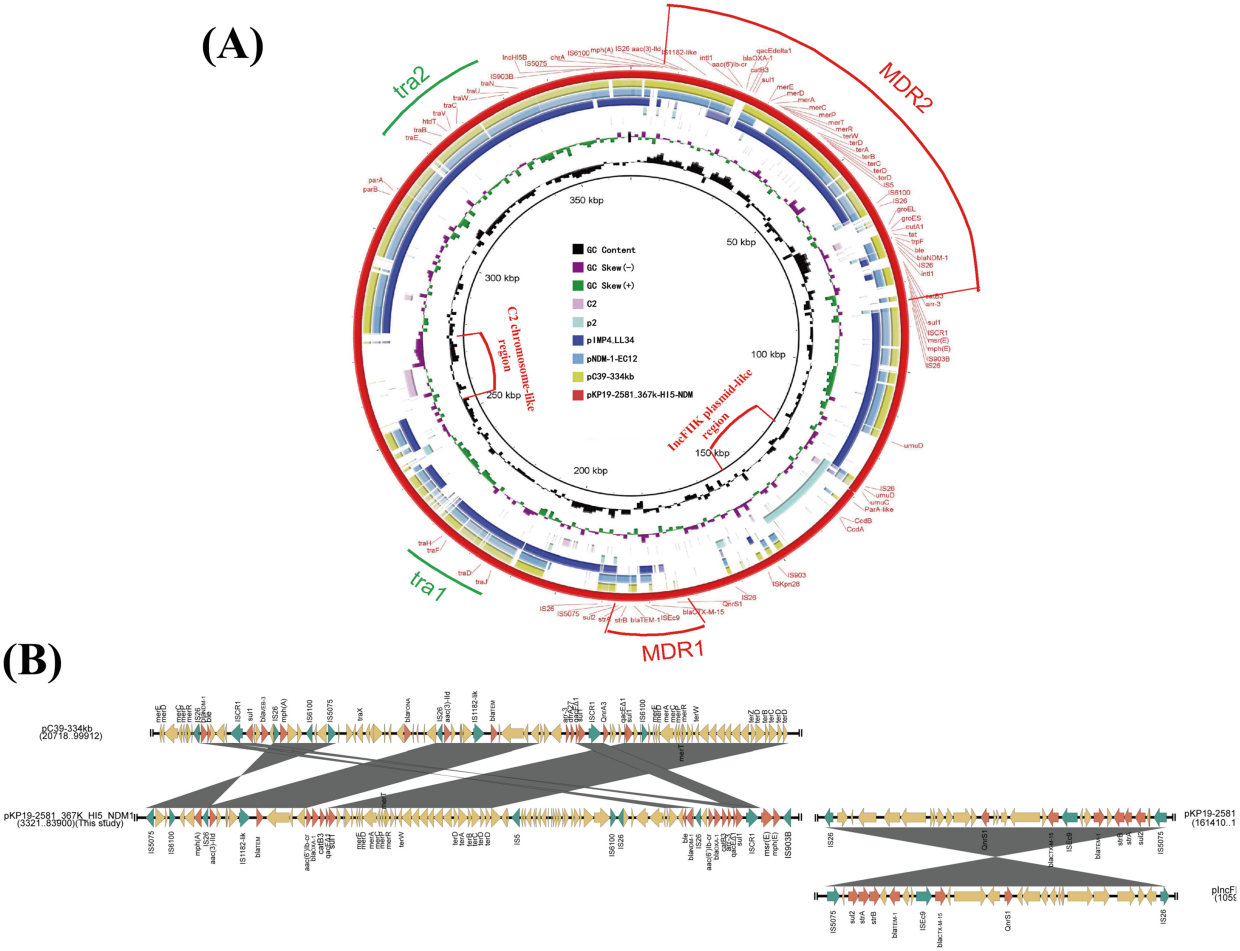
**(A)** Circular comparison between the plasmid pKP19-2581-367k-HI5-NDM1 and other reported similar plasmids. The circular map was generated using the BLAST Ring Image Generator (BRIG). **(B)** Linear alignment the MDR regions of plasmid pKP19-2581-367k-HI5-NDM1 with MDR region of plasmids pC39-334kb and pIncFIIK-IncFII. The linear map was generated using EasyFig.

Plasmid pKP19-2581-367k-HI5-NDM1 (CP120875), which contains a typical IncHI5-type backbone region and two separated multidrug-resistant regions. An online BLAST of the NCBI nr database displayed that pKP19-2581-367k-HI5-NDM1 shared a generally similar (81% query coverage and 99% nucleotide identity) IncHI5 backbone with plasmids pC39-334kb and pNDM-1-EC12 (Figure 1A). It is noteworthy that two additional regions which have never been described on IncHI5 plasmid were found to be inserted into the backbone region of pKP19-2581-367k-HI5-NDM1. The ~28-kb IncFIIk plasmid p2 (OW968065)-derived region harboring a toxin-antitoxin system CcdAB flanked by the IS*26* and IS*Kpn28* elements was inserted upstream of phage integrase, and other ~17-kb exogenous segment located immediately between two hypothetical protein shares similarity (38% query coverage and 80.96% nucleotide identity) to its corresponding fragment in the chromosome of *Enterobacteriaceae* strain C2 (CP053730) (Figures 1A, B). Insertion of segments originated from plasmid other than IncHI5 plasmid and chromosome suggested that the formation of pKP19-2581-367k-HI5-NDM1 has undergone complex combination processes based on structure of IncHI5-type plasmid.

Two MDR regions namely MDR1 and MDR2, were detected in the variable region of the plasmid pKP19-2581-367k-HI5-NDM1. The ~29-kb MDR1 contained multiple resistance genes including *qnrs1*, *bla*_CTX−M−15_, *bla*_TEM−1_, *strAB* and *sul2* exhibited 100% homologous to plasmid pIncFIBK-IncFII (CP084503) from *K. pneumoniae* (Figures 1A, B). Surprisingly, *bla*_NDM−1_ and two copies of *bla*_OXA−1_ genes were observed on MDR2. Detailed linear analysis showed that MDR2 was ~80kb in length, contained abundant AMR genes and mobile elements, and had high homology (query coverage 80%, nucleotide homology 99.99%) with the corresponding fragment of the *K. pneumoniae* IncHI5-like plasmid pC39-334kb (CP061701). Interestingly, the main difference between pKP19-2581-367k-HI5-NDM1 and pC39-334kb was in the composition of ARGs (Figure 1B). Compared with the MDR region of pC39-334kb, the highly mosaic MDR2 obviously underwent more complex and unique events of insertion, deletion or integration of AMR genes and different mobile elements. These findings jointly highlight the evolutionary potential of IncHI5 plasmids, as they may lead to the formation of new multidrug-resistant plasmids and become a reservoir of multidrug-resistant genes.

**Figure 2:**
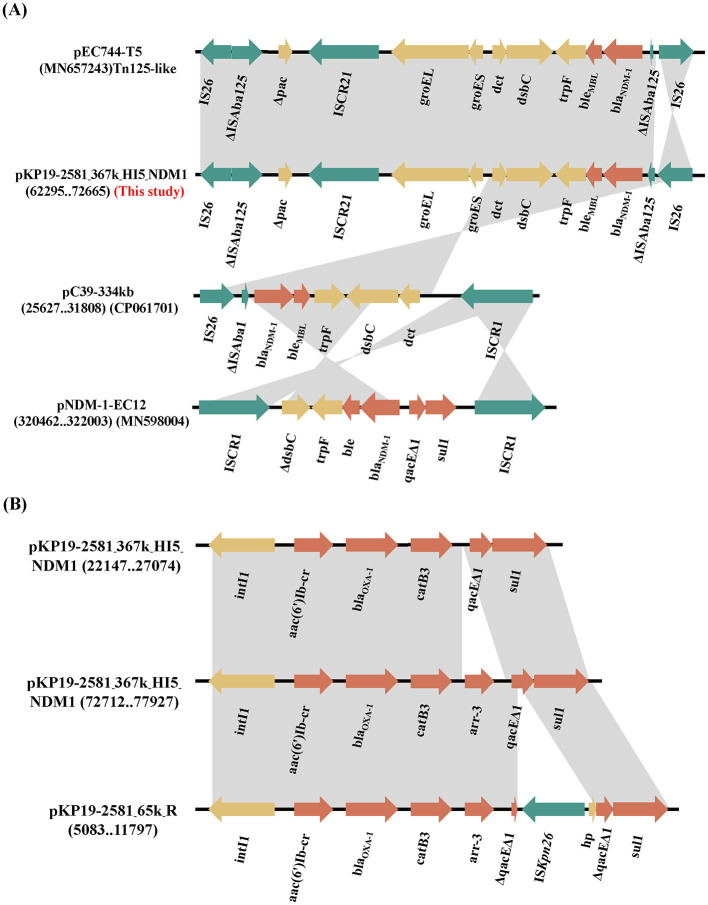
**(A)** Linear comparison of the genetic environment of *bla*_NDM−1_ among plasmids pKP19-2581-367k-HI5-NDM1and pEC744-T5, pC39-334kb, pNDM-1-EC12. **(B)** Linear comparison of genetic environment of *bla*_OXA−1_ among pKP19-2581-367k-HI5-NDM1 and pKP19-2581-65K-R. The plasmid comparison figure was generated by EasyFig.

Detailed genetic background analysis showed that the *bla*_NDM−1_ gene located on a previously described Tn*125-like* transposon structure (IS*26*-ΔIS*Aba125*-Δ*pac*-IS*CR21*-*groL*-*groES*-*dct*-*tat*-*iso*-*ble*_MBL_-*bla*_NDM−1_-ΔIS*Aba125*-IS*26*) (Weber et al., 2019) flanked by two IS*26* in the same direction in pKP19-2581-367k-HI5-NDM1 (Figure 2A). Compared with the *bla*_NDM−1_ genetic environment on the IncHI5 plasmid reported previously (CP061701 and MN598004), pKP19-2581-367k-HI5-NDM1 harbors a more complete Tn*125* structure and carries a broader array of ARGs. In addition, two copies of *bla*_OXA−1_ were found in the class 1 integron *intI1*-*aac(6*′*)-Ib-cr*-*bla*_OXA−1_-*catB3*-*qacE*Δ*1*-*sul1* and *intI1*-*aac(6*′*)-Ib-cr*-*bla*_OXA−1_-*catB3*-*arr-3*-*qacE*Δ*1*-*sul1*, which differ by the *arr-3* gene (Figure 2B). Interestingly, the IncR plasmid pKP19-2581-65K-R in *K. pneumoniae* KP19-2581 also carries a *bla*_OXA−1_ gene located in the class 1 integron, and an IS mobile element IS*Kpn26* was added (Figure 2B). As previously reported, mobile integrators (MIs) are ubiquitous elements that provide a platform for the acquisition, recombination, and expression of gene cassettes, many of which are antibiotic resistance genes (Recchia and Hall, 1995; Escudero et al., 2015). These elements are commonly associated with transposons and conjugative plasmids and play an important role in the evolution of resistance in pathogenic bacteria (Partridge et al., 2018). Therefore, the formation of pKP19-2581-367k-HI5-NDM1 may be the insertion of Tn*125-like* transposons mediated by IS*26* elements and a *bla*_OXA−1_-carrying class 1 integron from IncR plasmid pKP19-2581-65K-R from the same strain. Moreover, *bla*_OXA−1_ gene replicates in MDR 2 during the recombination process.

### Molecular characterization of an *mcr-8.1*-carrying IncFII/FIA plasmid

**Figure 3:**
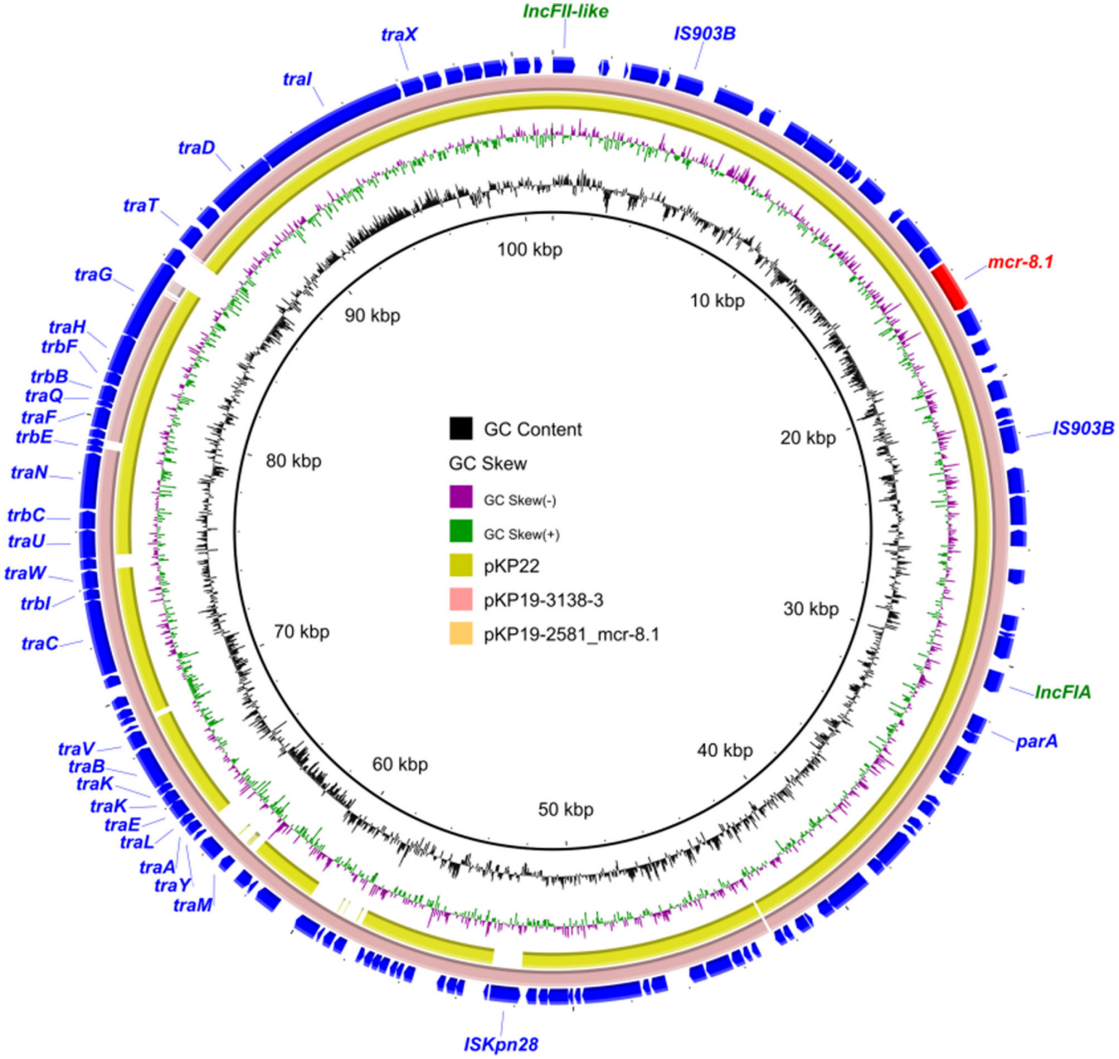
Circular comparison between the plasmid pKP19-2581-mcr-8.1 and other reported similar plasmids.

We found that *K. pneumoniae* strain KP19-2581 contained an *mcr*-*8.1*-harboring plasmid namely pKP19-2581-101k-mcr-FII-FIA (Figure 3). Replicon typing showed that the *mcr*-*8.1* gene was located on an IncFIA/IncFII plasmid, which had a length of 101,385 bp, with an average GC content of 51%, encoding 311 predicted ORFs. A BLASTN search against the NCBI nucleotide database revealed that the *mcr-8.1*-carrying plasmid pKP19-2581-101k-mcr-FII-FIA displayed 97% query coverage and 99.28% nucleotide identity to plasmid pKP19-3138-3 (CP090619) from the *K. pneumoniae*, 92% query coverage and 98.84% nucleotide identity to plasmid pKP22 (OL804390) from the *K. pneumoniae*. Analysis of the surrounding genetic context of the *mcr-8.1* gene in pKP19-2581-101k-mcr-FII-FIA showed the transfer of the *mcr-8.1* gene was presumably mediated by two IS*903B* in the same direction, constructing a mobile unit of IS*903B*-*orf*-*mcr*-*8.1*-*orf*-IS*903B*, as previously reported (Wu et al., 2020).

## Discussion and conclusion

There have been several reports of the concurrent presence of *bla*_NDM−1_ and *bla*_OXA−1_ in isolates of *Enterobacteriaceae* (Gondal et al., 2020; Xie et al., 2017). In the present study, strain KP19-2581 harbored one *bla*_NDM−1_ gene and two copies of the *bla*_OXA−1_ gene within the same MDR region on the IncHI5-like plasmid pKP19-2581-367k-HI5-NDM1. Notably, IncHI5 plasmids have garnered significant attention in recent years due to their ability to carry multiple antibiotic resistance genes. An increasing number of studies have identified carbapenem resistance genes on IncHI5 plasmids (Liang et al., 2018; Joseph et al., 2020; Chen et al., 2021; Liu et al., 2021; Wang et al., 2021; Zhang et al., 2021; Dong et al., 2022; Luo et al., 2023), highlighting their role as key reservoirs for resistance genes, particularly carbapenemase genes.

The ISs, which can autonomously replicate and insert into various genomic locations, play a pivotal role in gene recombination, mutation, and the evolution and dissemination of plasmids (Partridge et al., 2018). In our study, the insertion of multiple IS*26* elements mediated several insertion and rearrangement events involving a large number of ARGs, including the *bla*_NDM−1_-carrying MDR region, thus accelerating the evolution of IncHI5-like plasmids.

Notably, the emergence of the colistin resistance gene *mcr-8.1* has further weakened the efficacy of treatments for infections caused by multi-drug-resistant bacteria. When the IncHI5-like plasmid and the *mcr-8.1* coexist in the same bacterial strain, clinical treatment will face severe challenges and is highly likely to fall into the difficult situation of having “no drugs available”. Moreover, considering that the IncHI5 plasmid has a broad host adaptability, it has aggravated the global epidemic risk of drug resistance (Liang et al., 2018). Therefore, in such circumstances, it is necessary to strengthen monitoring and control measures to prevent the spread of drug-resistant strains and the outbreak of infections.

The oriTfinder showed relevant genes on this plasmid, yet the oriT region was absent. OriT is crucial for plasmid transfer. Since it's necessary for conjugative plasmids, an incomplete MOB module might explain pKP19-2581-367k-HI5-NDM1's non-conjugative nature. In addition, the plasmid harbors multiple antibiotic resistance genes, and its acquisition may impose a substantial fitness cost on the host strain, thereby limiting its ability to be transferred to other bacterial recipients (Liu et al., 2024). Surprisingly, the first appearance of the IncHI5-like plasmid could be traced back to 2013. Over the past decade, it has not experienced rapid growth, largely due to the fact that only a tiny minority of Inchi5-like plasmids are capable of undergoing conjugative transfer (Luo et al., 2023). However, due to the co-location of genes related to heavy metal resistance and other non-carbapenem resistance genes in IncHI5-like plasmids, stubborn selection pressure may also contribute to the maintenance and spread of IncHI5-like plasmids (Baker-Austin et al., 2006).

In summary, this study is the first to report an *mcr-8.1*-positive *K. pneumoniae* strain co-harboring *bla*_NDM−1_ and two copies of *bla*_OXA−1_ within an IncHI5-like plasmid. The structure of plasmid, comprising three distinct regions—an IncHI5 plasmid-like region, an IncFIIk plasmid-like region, and a C2 chromosome-like region—highlights the strong evolutionary potential of IncHI5 plasmids. The emergence of IncHI5-like plasmids carrying multiple carbapenemase genes underscores their potential to become significant vectors for carbapenemase gene transmission. Although these plasmids are typically non-conjugative, the presence of abundant AMR genes and co-selection pressures may sustain their persistence, further facilitating the dissemination of high-level carbapenemase resistance among clinical isolates. Continuous monitoring of the evolutionary dynamics and spread of IncHI5-like plasmids is therefore imperative to mitigate their potential impact on public health.

## Data Availability

The datasets presented in this study can be found in online repositories. The names of the repository/repositories and accession number(s) can be found in the article/supplementary material.
