# Supplementary material for: Identification of an IncHI5-like plasmid co-harboring blaNDM−1 and blaOXA−1 in mcr-8.1-positive Klebsiella pneumoniae isolate
Source: Front Microbiol. 2025 May 27;16:1601035. doi: 10.3389/fmicb.2025.1601035 (PMC12149150; doi:10.3389/fmicb.2025.1601035)
Supplement: Supplementary file 1 [file Table_1.docx]

**Table S1. Primers in this study.**

| Gene name | Primers | Sequence (5'-3') | Tm（℃） | Length（bp） |
| --- | --- | --- | --- | --- |
| *bla*_NDM_ | bla_NDM_-F | GCAGCTTGTCGGCCATGCGGGC | 60 | 782 |
|  | *bla*_NDM_-R | GGTCGCGAAGCTGAGCACCGCAT |  |  |
| *bla*_IMP_ | *bla*_IMP_-F | GAAGGCGTTTATGTTCATAC | 60 | 587 |
|  | *bla*_IMP_-R | GTACGTTTCAAGAGTGATGC |  |  |
| *bla*_VIM_ | bla_VIM_-F | GTTTGGTCGCATATCGCAAC | 60 | 389 |
|  | *bla*_VIM_-R | AATGCGCAGCACCAGGATAG |  |  |
| *bla*_KPC_ | *bla*_KPC_-F | TGTCACTGTATCGCCGTC | 60 | 900 |
|  | *bla*_KPC_-R | CTCAGTGCTCTACAGAAACC |  |  |
| *bla*_OXA_ | *bla*_OXA_*-*F | GCGTGGTTAAGGATGAACAC | 60 | 438 |
|  | *bla*_OXA_*-*R | CATCAAGTTCAACCCAACCG |  |  |
| *mcr-8* | *mcr*-F | ATCAGCCAAACCTATCCCATCG | 55 | 564 |
|  | *mcr*-R | GCAGACGCACAGCAATGCCTAT |  |  |

**Table S2. Antimicrobial susceptibilities of the MDR *Klebsiella pneumoniae* KP19-2581.**

| Antimicrobial category | Antimicrobial  agents | MIC*^a^* (μg/mL) |
| --- | --- | --- |
|  |  | KP19-2581 |
| Penicillins | Ampicillin | >256 *^b^* |
| Antipseudomonal penicillins with b-lactamase inhibitors | Piperacillin/  tazobactam | >1024 |
| Nonextended spectrum cephalosporins | Cefazolin | >256 |
| Extended-spectrum cephalosporins | Ceftazidime | >64 |
|  | Cefoxitin | >256 |
| Carbapenems | Meropenem | 16 |
| Monobactams | Aztreonam | >64 |
| Aminoglycosides | Gentamicin | 128 |
|  | Amikacin | 4 |
| Phenicols | Chloroamphenicol | >128 |
| Tetracyclines | Doxycycline | 8 |
|  | Tetracycline | 8 |
| Glycylcyclines | Tigecycline | 2 |
| Polymyxin C | Colistin | 2 |

^a^ MIC, minimum inhibitory concentration.

^b^ Resistance to antimicrobial agents showed in bold.
